# Antiproteinuric Effect of Sparsentan in Patients with Genetic-Associated FSGS Enrolled in the DUPLEX Trial

**DOI:** 10.2215/CJN.0000000948

**Published:** 2025-12-23

**Authors:** Jennifer Yee, Wu Gong, Jula Inrig, Michelle N. Rheault, Angela J. Gruber, Patricia W. Bedard, Radko Komers, Howard Trachtman

**Affiliations:** 1 Division of Nephrology, Department of Pediatrics, University of Michigan, Ann Arbor, Michigan; 2 Travere Therapeutics, Inc., San Diego, California; 3 Department of Pediatrics, University of Minnesota Medical School, Minneapolis, Minnesota; 4 PreventionGenetics, Marshfield, Wisconsin

**Keywords:** glomerular disease, glomerulosclerosis, randomized controlled trials, genetic kidney disease

## Abstract

**Key Points:**

- Sparsentan lowered proteinuria, with more frequent complete remission of proteinuria versus irbesartan in patients with genetic forms of FSGS.
- Fewer patients with genetic forms of FSGS progressed to the composite kidney endpoint with sparsentan versus irbesartan.
- Consistent with the overall DUPLEX population, the results support sparsentan's antiproteinuric benefit in patients with genetic forms of FSGS.

**Background:**

FSGS is a podocytopathy that is diagnosed based on characteristic histopathologic lesions. Certain forms of FSGS with underlying genetic variants associated with the disease, including variants in podocyte proteins, as well as apolipoprotein L1 (*APOL1*) risk alleles, and variants in collagen type 4 *α*3, *α*4, and *α*5 (*COL4A3–5*) proteins, are typically resistant to current treatments.

**Methods:**

The DUPLEX clinical trial assessed the efficacy and safety of sparsentan, a dual endothelin angiotensin receptor antagonist, in patients with biopsy-proven or genetic FSGS (gFSGS) and demonstrated a greater antiproteinuric effect over 108 weeks compared with the active control irbesartan. This *post hoc*, exploratory analysis assessed the efficacy of sparsentan in the subset of patients enrolled in DUPLEX who had pathogenic variants in genes coding for podocyte proteins, *COL4A3–5* variants, and *APOL1* high-risk genotypes, all together referred to as gFSGS.

**Results:**

Next-generation sequencing identified 31 patients with podocyte gene variants, 25 with *COL4A3–5* gene variants, and 14 with *APOL1* high-risk genotypes. Baseline characteristics varied between genetic subgroups, with slightly younger patients, on average, in the podocyte gene variant group, and more African American patients in the *APOL1* high-risk genotype group. In this exploratory analysis, sparsentan treatment resulted in substantial and sustained proteinuria reductions and numerically more frequent complete remission of proteinuria compared with irbesartan, consistent with observations in the full DUPLEX study population. Moreover, a lower proportion of sparsentan-treated versus irbesartan-treated patients reached composite kidney endpoints.

**Conclusions:**

These findings support sparsentan's antiproteinuric benefit in patients with gFSGS, a subgroup that is often resistant to other therapeutic interventions.

**Clinical Trial registry name and registration number:**

Clinicaltrials.gov, NCT03493685.

## Visual Abstract

**Figure:**
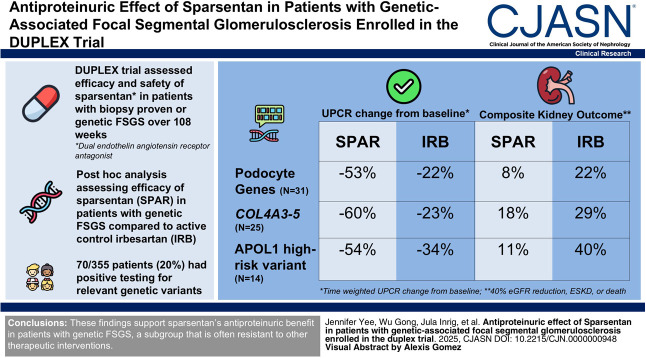

## Introduction

FSGS is a rare, progressive, kidney condition with a pathognomonic histologic pattern of podocyte and glomerular injury.^1,2^ According to Kidney Disease Improving Global Outcomes guidelines,^1,3^ it can be classified as: (*1*) primary FSGS that is presumed to be immunologically mediated; (*2*) genetic FSGS (gFSGS); (*3*) secondary FSGS, with underlying causes including reduced kidney mass, medications, infections, or conditions such as obesity, hypertension, lupus nephritis, or diabetes; and (*4*) FSGS of unknown cause.

gFSGS is most often the result of variations in podocyte genes that encode proteins in the actin cytoskeleton, cell metabolism, cell adherence and migration, extracellular matrix components, nuclear pores, and intracellular transport.^4^ In general, gFSGS is refractory to corticosteroids and most available second-line immunosuppressive treatments and affected patients are at high risk of progression to kidney failure, often at a very young age. The lack of effective treatments highlights the urgent unmet need for better supportive or targeted therapies in this patient subgroup.

In addition to genetic variations in podocyte proteins, polymorphisms in the gene encoding apolipoprotein L1 (*APOL1*) are associated with a higher risk for kidney disease. Two specific high-risk variants, termed G1 and G2, are relatively common among individuals of African ancestry and display strong linkage with the occurrence of certain kidney diseases, including FSGS, HIV nephropathy, and lupus nephritis.^5,6^ In patients with some forms of CKD, including FSGS, the presence of two *APOL1* high-risk alleles is associated with more rapid disease progression and a higher risk of kidney failure.^7,8^

Finally, genetic studies in a broad spectrum of patients with proteinuric forms of CKD have documented a high prevalence of collagen type 4 *α*3, *α*4, and *α*5 genetic variants (*COL4A3–5*).^9,10^ There is increasing evidence that these variants are relatively common in patients with CKD and histologic evidence of FSGS.^11–13^ These genetic abnormalities may contribute to the pathogenesis of the disease and/or rate of progression as the primary cause or disease modifiers.^6^ However, there is a paucity of data on the optimal treatment of patients with FSGS and *COL4A3–5* variants.

The DUPLEX trial evaluated the efficacy and safety of sparsentan versus maximum labeled dose irbesartan in 371 patients with biopsy-proven FSGS or documentation of a genetic mutation associated with FSGS, excluding those with known secondary causes. Sparsentan is a dual endothelin angiotensin receptor antagonist that blocks the endothelin-1 and angiotensin II pathways, key mediators of podocyte damage and kidney injury in FSGS.^14–16^ Overall, the findings indicated a significantly greater and sustained proteinuria reduction with sparsentan compared with the active control irbesartan.^17^ This *post hoc* analysis assessed sparsentan's efficacy on lowering proteinuria in the subset of patients enrolled in DUPLEX with gFSGS, including variants in podocyte proteins, *COL4A3–5*, or high-risk *APOL1* alleles.

## Methods

### Patients and Study Design

The design and overall results of the DUPLEX trial (NCT03493685; 2018-04-03) have been published previously.^17–19^ In brief, patients aged 8–70 years with biopsy-proven FSGS, with or without documentation of a disease-associated variant in genes coding for podocyte proteins, eGFR of >30 ml/min per 1.73 m^2^, and urine protein-to-creatinine ratio (UPCR) of >1.5 g/g were enrolled. They were randomized (1:1) to receive sparsentan or irbesartan for 108 weeks. The interim efficacy endpoint was the FSGS partial remission endpoint (UPCR of ≤1.5 g/g and >40% UPCR reduction from baseline),^20^ and the primary efficacy endpoint was the eGFR slope analyzed after completion of the double-blind treatment period. Outcome analyses also examined changes in proteinuria (percentage reduction; achievement of complete remission of proteinuria [CR, defined as UPCR of <0.3 g/g at any time]) and the proportion of patients reaching 40% eGFR reduction or kidney failure (eGFR of <15 ml/min per 1.73 m^2^, dialysis, or transplant) with sparsentan versus irbesartan.

Participants were enrolled following institutional review board or ethics committee approvals at each investigational site in accordance with Good Clinical Practice and the Declaration of Helsinki. All participants provided written informed consent before study enrollment.

### Identification of Genetic Cases

Next-generation sequencing on a 73-gene FSGS panel (PreventionGenetics, Marshfield, WI; Supplemental Table 1) was completed for 355 of 371 study participants. Variants were interpreted according to PreventionGenetics and the American College of Medical Genetics and Genomics standards and guidelines.^21^ Patients with pathogenic or likely pathogenic variants in podocyte genes were classified as having gFSGS with Mendelian inheritance and analyzed as a distinct group of podocyte gene variants. In addition, due to reports that the pathogenicity of the podocin (*NPHS2*) variant p.Arg229Gln is dependent on the location of the second variant in the gene,^22,23^ patients who had compound heterozygous *NPHS2* variants, including p.Arg229Gln, were classified as having gFSGS only if the other pathogenic or likely pathogenic variant is located in exon 7 or 8 of *NPHS2*. Considering the evidence from studies in large cohorts of patients with CKD showing a high prevalence of *COL4A3–5* variants,^24,25^ pathogenic or likely pathogenic variants in genes coding for type 4 collagen proteins (*COL4A3*, *COL4A4*, *COL4A5*) were also included but analyzed as a separate *COL4A3–5* variant group. The *APOL1* risk alleles (G1: rs73885319 and rs60910145 and G2: rs71785313) were identified from the PreventionGenetics genetic panel, and patients were classified as having *APOL1* high-risk genotypes if they have two *APOL1* risk alleles (G1/G1, G1/G2, G2/G2).^26^ To ensure that the analysis was not confounded by heterogeneous forms of kidney disease, patients with podocyte gene variants, *COL4A3–5* genetic variants, and *APOL1* high-risk genotypes were analyzed separately. Patients with mosaic copy number variants or trisomy X were excluded from the analysis.

### Statistical Analyses

*Post hoc* descriptive analyses were conducted to characterize baseline demographics and clinical features and to explore treatment responses across gFSGS subgroups. Continuous variables were summarized using means with SDs and medians with interquartile ranges, while categorical variables were reported as counts and percentages. Geometric least-squares mean percentage changes were calculated using natural log-transformed proteinuria in a mixed model with repeated measures (MMRM) as specified in the DUPLEX study design^18^ to show the percent difference in UPCR from baseline at each visit. As UPCR can have large values, the log of UPCR ratio, log(UPCR at visit/UPCR at baseline), was modeled as an outcome in the MMRM. The results were back-transformed as percentage change, or percentage reduction, as (exp[least-squares mean of log(UPCR ratio)]−1)×100. Per the study's statistical analysis plan, a first-order autoregressive structure was applied for this subgroup analysis because the MMRM with prespecified covariates and unstructured covariance matrix failed to converge. Time-weighted UPCR changes over the double-blind period were estimated using weighted least-squares means from the MMRM model through the trapezoidal rule.

Although these analyses were exploratory in nature, we report changes from baseline with corresponding measures of variability (*e.g*., 95% confidence intervals [CIs]) and time-averaged proteinuria data with corresponding *P* values to express uncertainty and facilitate hypothesis generation. Baseline characteristics are summarized using descriptive statistics only. All statistical procedures were performed using SAS version 9.4.

## Results

Altogether, we report data from 70 patients in whom genetic variants could contribute to the development and/or progression of FSGS. Owing to the distinct nature of the genetic variants and the considerable heterogeneity of these patients, we conducted separate analyses of data in those with podocyte variants, *COL4A3–5* variants, and *APOL1* high-risk genotypes and did not perform a pooled analysis.

The podocyte genes with variants identified in DUPLEX are listed in Table 1. Of 355 participants in DUPLEX for whom next-generation sequencing was completed, 31 (9%) were identified as having gFSGS with Mendelian inheritance related to a podocyte protein, 13 of whom were assigned to sparsentan and 18 to irbesartan (Table 2). Most had recessive *NPHS2* disease (approximately 50% among patients with a podocyte gene variant). In addition, 25 patients (7%) were identified to have *COL4A3–5* genetic variants (genotype and sex distributions are presented in Supplemental Table 2), of whom 11 received sparsentan and 14 received irbesartan. Finally, 14 patients (4%) had two *APOL1* high-risk alleles, nine in the sparsentan and five in the irbesartan groups. None of the patients in the podocyte or *COL4A3–5* groups had compound heterozygous or homozygous *APOL1* high-risk alleles; thus, there was no patient overlap in the three main genetic subgroups in our analyses. Of note, one patient with a heterozygous *APOL1* G2 risk allele existed in the podocyte gene variant group and one additional patient with a heterozygous *APOL1* G1 risk allele was identified in the *COL4A3–5* group.

**Table 1:** Podocyte gene variants in DUPLEX by the treatment group

Gene	Sparsentan	Irbesartan	Total
*NPHS2*	9	6	15
*CD2AP*	0	1	1
*INF2*	1	3	4
*LMX1B*	1	3	4
*NPHS1*	1	0	1
*TRPC6*	0	2	2
*WT1*	1	3	4

*CD2AP*, CD2-associated protein; *INF2*, inverted formin 2; *LMX1B*, LIM homeobox transcription factor 1*β*; *NPHS1*, nephrin; *NPHS2*, podocin; *TRPC6*, transient receptor potential cation channel subfamily C member 6; *WT1*, WT1 transcription factor.

**Table 2:** General and baseline characteristics of patients by genetic and treatment groups

Patient Characteristics	Podocyte Gene Variants				*COL4A3–5* Variants		*APOL1* High-Risk Genotypes	
	All		Recessive *NPHS2* Disease					
	Sparsentan (*n*=13)	Irbesartan (*n*=18)	Sparsentan (*n*=9)	Irbesartan (*n*=6)	Sparsentan (*n*=11)	Irbesartan (*n*=14)	Sparsentan (*n*=9)	Irbesartan (*n*=5)
Age at informed consent, yr, mean (SD)	26 (13)	36 (15)	26 (15)	32 (13)	41 (12)	42 (11)	36 (13)	32 (22)
**Age group, yr, *n* (%)**								
<18	6 (46)	2 (11)	4 (44)	1 (17)	0 (0)	0 (0)	1 (11)	2 (40)
≥18	7 (54)	16 (89)	5 (56)	5 (83)	11 (100)	14 (100)	8 (89)	3 (60)
**Sex, *n* (%)**								
Male	4 (31)	5 (28)	3 (33)	2 (33)	5 (45)	6 (43)	5 (56)	3 (60)
Female	9 (69)	13 (72)	6 (67)	4 (67)	6 (55)	8 (57)	4 (44)	2 (40)
**Race, *n* (%)^a^**								
Asian	0 (0)	1 (6)	0 (0)	0 (0)	1 (9)	3 (21)	0 (0)	0 (0)
Black or African American	2 (15)	1 (6)	2 (22)	1 (17)	0 (0)	1 (7)	7 (78)	2 (40)
Other	0 (0)	1 (6)	0 (0)	0 (0)	0 (0)	0 (0)	0 (0)	3 (60)
White	12 (92)	15 (83)	8 (89)	5 (83)	10 (91)	10 (71)	2 (22)	0 (0)
BMI, kg/m^2^, mean (SD)	24 (5)	25 (5)	24 (5)	23 (4)	29 (6)	26 (5)	34 (7)	34 (9)
**BMI group, kg/m** ^**2**^ **, *n* (%)**								
<27	9 (69)	11 (61)	6 (67)	4 (67)	5 (46)	8 (62)	1 (11)	1 (20)
≥27	4 (31)	7 (39)	3 (33)	2 (33)	6 (55)	5 (39)	8 (89)	4 (80)
**eGFR, ml/min per 1.73 m** ^**2**^								
Mean (SD)^b^	75 (43)	74 (47)	80 (42)	109 (69)	54 (26)	53 (20)	59 (26)	47 (8)
Median (IQR)	56 (47, 93)	62 (46, 75)	79 (53, 93)	123 (32, 141)	47 (40, 67)	46 (40, 55)	50 (41, 66)	44 (42, 50)
**UPCR, g/g**								
Mean (SD)	4.9 (2.4)	4.2 (2.3)	5.0 (2.9)	4.5 (3.1)	3.2 (1.5)	2.8 (1.5)	2.7 (1.8)	2.7 (1.6)
Median (IQR)	4.5 (3.5–5.3)	3.7 (2.9–5.2)	3.7 (3.5–5.7)	3.9 (2.9–6.7)	2.6 (2.0–4.2)	2.6 (1.9–4.5)	1.8 (1.6–3.2)	2.1 (1.9–2.3)

*APOL1*, apolipoprotein L1; *COL4A3–5*, collagen type 4 *α*3, *α*4, and *α*5; BMI, body mass index; CKD-EPI, CKD Epidemiology Collaboration; IQR, interquartile range; *NPHS2*, podocin; UPCR, urine protein-to-creatinine ratio.

a Race was reported by the patients; patients could have selected more than one race.

b eGFR was determined using the CKD Epidemiology Collaboration equation for patients ≥16 years of age and the modified Schwartz formula for patients <16 years of age at screening.

Baseline characteristics by subgroup are presented in Table 2. On average, patients with podocyte gene variants were younger than those with *COL4A3–5* variants or *APOL1* high-risk genotypes (32±15 versus 41±11 or 35±16 years, respectively), particularly those with recessive *NPHS2*-related FSGS (28±14 years). There was no patient younger than 18 years with a *COL4A3–5* variant. By contrast, patients with *APOL1* high-risk genotypes were more frequently ≥18 years. Interestingly, more patients with *APOL1* high-risk genotypes had a higher body mass index (BMI) than those in the remaining genetic groups (34±7 versus 25±5 and 28±5 in the podocyte gene and *COL4A3–5* variant groups, respectively). Most patients were White except in the *APOL1* high-risk group, which included nine (64%) African American patients. In addition, patients in both the *COL4A3–5* variant and *APOL1* high-risk groups had, on average, lower baseline eGFR than those with podocyte gene variants (54±22 and 55±22 versus 74±44 ml/min per 1.73 m^2^, respectively). While patients typically had nephrotic-range proteinuria, patients in the podocyte gene variant group were observed to have higher baseline UPCR levels than those in the other two genetic groups (median, 3.8 versus 2.6 and 2.0 g/g in the *COL4A3–5* variant and *APOL1* high-risk groups, respectively).

**Figure 1:**
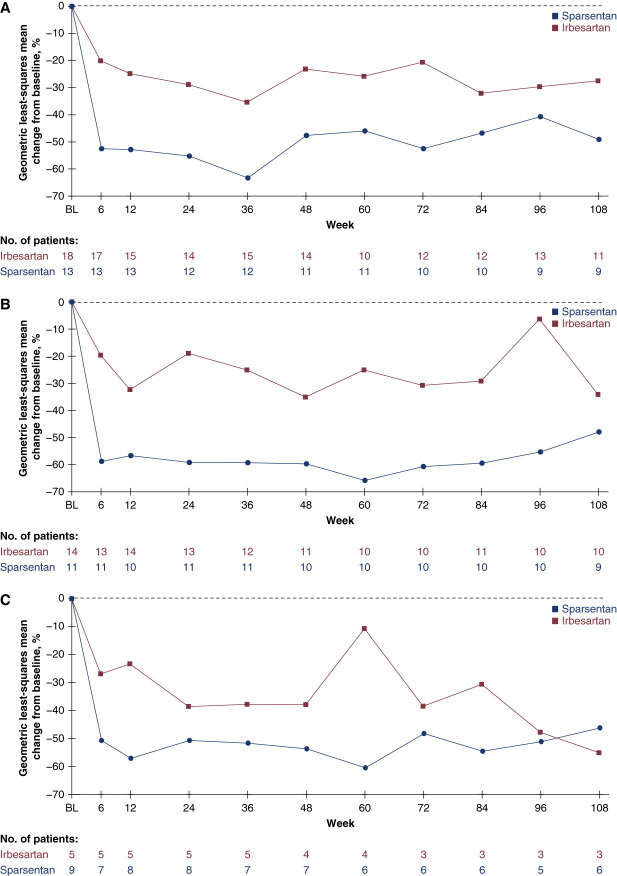
**Effect of sparsentan compared with irbesartan on UPCR.** Percent changes from BL in UPCR at each study visit in (A) patients with pathogenic or likely pathogenic variants in genes coding for podocyte proteins, (B) patients with *COL4A3–5* variants, and (C) patients with *APOL1* high-risk genotypes. *APOL1*, apolipoprotein L1; BL, baseline; *COL4A3–5*, collagen type 4 *α*3, *α*4, and *α*5; UPCR, urine protein-to-creatinine ratio.

The geometric least-squares mean percentage change from baseline in UPCR by subgroup is presented in Figure 1 and Supplemental Table 3. In patients with podocyte gene variants, sparsentan resulted in a more rapid and pronounced proteinuria reduction compared with irbesartan, with the greatest difference observed at week 6 (Figure 1A; week 6: −53% versus −20%). This effect was sustained over the treatment period, although the between-treatment arm difference modestly narrowed after week 12. A similar trend for UPCR reduction was observed in a subset of 15 patients with *NPHS2* variants (Supplemental Figure 1). A similar but more pronounced pattern was observed in patients with *COL4A3–5* gene variants (Figure 1B). Sparsentan treatment resulted in profound (approximately 50%) sustained reduction of UPCR in the patients with *APOL1* high-risk genotypes, although the treatment effect versus irbesartan was not sustained during the second year of the treatment period (Figure 1C).

In addition to determination of the change in UPCR from baseline at each visit, similar to previously reported analyses from DUPLEX,^17^ we also analyzed time-weighted UPCR change from baseline. The results (Table 3) further support the greater antiproteinuric effect of sparsentan, with a significant treatment effect demonstrated in patients with podocyte (relative reduction of 32.5% [95% CI, 3.9 to 52.7; *P* = 0.03]) and *COL4A3–5* variants (relative reduction of 44.9% [95% CI, 16.5 to 63.6; *P* = 0.01]).

**Table 3:** Time-weighted urine protein-to-creatinine ratio change from baseline by genetic and treatment groups

Type of Variant/Genotype	Time-Averaged Change from Baseline, % (95% CI)		Relative Change, % (95% CI)	*P* Value
	Sparsentan	Irbesartan		
Podocyte gene variants	−53.4 (−63.7 to −40.3)	−21.5 (−36.8 to −2.6)	−32.5 (−52.7 to −3.9)	0.03
*COL4A3–5* variants	−59.7 (−70.3 to −45.5)	−23.1 (−41.8 to 1.6)	−44.9 (−63.6 to −16.5)	0.01
*APOL1* high-risk genotypes	−54.3 (−76.6 to −10.9)	−33.8 (−71.2 to 52.2)	−27.3 (−78.1 to 141.2)	0.57

*APOL1*, apolipoprotein L1; CI, confidence interval; *COL4A3–5*, collagen type 4 *α*3, *α*4, and *α*5.

Importantly, a numerically higher percentage of patients treated with sparsentan achieved CR compared with irbesartan across all three gFSGS groups. CR occurred exclusively in sparsentan-treated patients among those with podocyte gene variants (8% versus 0%), in 18% versus 7% of patients with *COL4A3–5* gene variants, and in 33% versus 20% of patients with *APOL1* high-risk genotypes.

**Figure 2:**
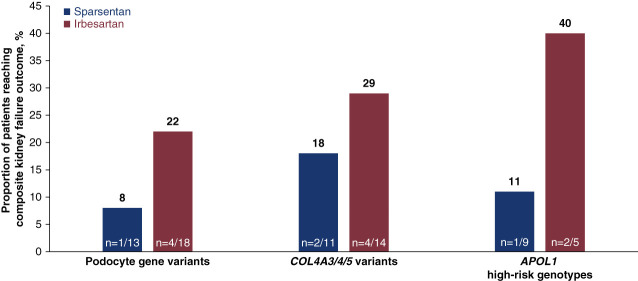
**Effect of sparsentan compared with irbesartan on the composite kidney endpoint.** Proportion of patients reaching the composite endpoint of 40% eGFR reduction, ESKD, or death.

The changes in eGFR from baseline to week 108 are presented in Table 4, showing no consistent pattern of eGFR decline across treatment groups within different genetic subgroups. However, patients with *APOL1* high-risk genotypes experienced numerically less absolute eGFR decline with sparsentan compared with irbesartan. In addition, the composite kidney outcome—defined as a 40% reduction in eGFR, progression to ESKD, or death—occurred numerically less frequently in patients receiving sparsentan versus irbesartan across all three gFSGS groups: 8% versus 22% among those with podocyte gene variants, 18% versus 29% among those with *COL4A3–5* variants, and 11% versus 40% among those with *APOL1* high-risk genotypes (Figure 2).

**Table 4:** Changes in eGFR by genetic and treatment groups

eGFR Change from Baseline to Week 108	Podocyte Gene Variants		*COL4A3–5* Variants		*APOL1* High-Risk Genotypes	
	Sparsentan (*n*=8)	Irbesartan (*n*=11)	Sparsentan (*n*=9)	Irbesartan (*n*=10)	Sparsentan (*n*=6)	Irbesartan (*n*=3)
Mean (SD), ml/min per 1.73 m^2^	−20 (29)	−17 (13)	−12 (11)	−10 (8)	−7 (6)	−11 (11)
Median (IQR), ml/min per 1.73 m^2^	−7 (−48 to −2)	−13 (−27 to −8)	−8 (−17 to −4)	−11 (−15 to −2)	−8 (−12 to −1)	−7 (−23 to −3)

*APOL1*, apolipoprotein L1; *COL4A3–5*, collagen type 4 *α*3, *α*4, and *α*5; IQR, interquartile range.

An additional 15 patients (4% of those sequenced) with one *APOL1* high-risk allele (*APOL1* heterozygous group) were identified (sparsentan, *n*=5; irbesartan, *n*=10). As presented in Supplemental Table 4, the age, BMI, and baseline eGFR of patients in the *APOL1* heterozygous group were intermediate between those of the podocyte gene variant group and *APOL1* high-risk group. In this subgroup, sparsentan resulted in greater proteinuria reduction versus irbesartan throughout the treatment period (Supplemental Table 4).

## Discussion

With the increasing accessibility of clinical genetic testing and more widespread implementation in nephrology, there are concomitant increases in the diagnosis of gFSGS. Given this emerging trend in practice, and more effective proteinuria reduction with sparsentan than irbesartan in the overall FSGS population,^17^ we conducted this *post hoc* analysis of the patients from the DUPLEX trial with gFSGS attributed to a genetic variant in a podocyte protein, *COL4A3–5*, or *APOL1* high-risk alleles to assess whether patients with gFSGS may also benefit from sparsentan.

The 9% prevalence of gFSGS due to podocyte gene variants in DUPLEX aligns with published estimates of the prevalence of monogenic podocyte gene variant causes of nephrotic syndrome ranging from 5% to 30% depending on age of onset^27–31^ and findings from the NEPTUNE cohort, which, like DUPLEX, included both pediatric and adult patients.^32^ DUPLEX included a high proportion of patients with *NPHS2* variants, consistent with previous studies identifying *NPHS2* variants as the most common podocyte gene abnormality.^33,34^ Patients with two pathogenic variants in *NPHS2* tend to develop childhood-onset FSGS,^23^ and in the DUPLEX trial, those with recessive *NPHS2*-related FSGS were indeed younger than those in the non-*NPHS2* genetic group. The detection rate of *COL4A3–5* gene variants is comparable with recent biopsy-proven FSGS case series, where 10%–40% of patients had these variants.^11,13,35,36^

*APOL1* high-risk alleles were present in nine of 29 African American patients in the trial, a higher prevalence than in the overall African American population, but consistent with other kidney disease studies.^5,37^ More patients with *APOL1* high-risk genotypes had a higher BMI than those in other groups, suggesting that patients with *APOL1* high-risk genotypes had different metabolic risk profiles.

We consistently observed a greater, more rapid, and sustained reduction of proteinuria with sparsentan versus irbesartan in patients with gFSGS due to variants in podocyte specific genes. Moreover, the relatively large number (*n*=15) of patients with an *NPHS2* variant enabled analysis of this particular subgroup, where sparsentan showed a more pronounced reduction in proteinuria, particularly at early time points. To the best of our knowledge, DUPLEX is the largest interventional study to date in this rare population. As there are no effective therapies for these patients, current data suggest sparsentan as a promising option for the reduction of severe proteinuria. In this context, we observed one patient with an nephrin (*NPHS1*) variant, traditionally considered unresponsive to therapy, who responded exceptionally well to sparsentan, achieving and maintaining CR.

A stronger antiproteinuric response to sparsentan versus irbesartan was also observed in the subgroup of patients with *COL4A3–5* gene variants. There is a possibility that *COL4A3–5* variants are a genetic modifier in some patients with FSGS rather than manifesting as typical Alport syndrome. Among patients with *COL4A3–5* gene variants, one male with a *COL4A5* gene variant and nephrotic-range proteinuria responded well to sparsentan, maintaining CR throughout treatment. Recent evidence indicates that modification of basement membrane components, including *COL4A3–5*, leads to altered localization and increased degradation of slit diaphragm proteins.^38^ This links the primary defect in *COL4A3–5* gene variants to a disturbance in the podocyte, the hallmark feature of FSGS. Our findings support the antiproteinuric benefit of sparsentan in a patient with a documented *COL4A3–5* gene variant and biopsy-confirmed FSGS. This observation offers the possibility of kidney function preservation with sustained long-term use.

*APOL1* high-risk genotypes are a significant risk factor for FSGS with a more rapid rate of progression.^7,8^ Consistent with observations in the other genetic subgroups, a substantial proteinuria reduction (>50%) with sparsentan was demonstrated over the follow-up period in *APOL1* high-risk patients. However, the treatment effect versus irbesartan did not persist into the second year of treatment, likely due to the reduced number of patients available for analysis. In addition, in *APOL1* high-risk patients, irbesartan also demonstrated a strong antiproteinuric effect, resulting in relatively smaller treatment effect of sparsentan versus irbesartan. The effect of irbesartan suggests an important role of angiotensin II type 1 receptor (AT_1_R) inhibition in lowering proteinuria in this patient subgroup. Nonetheless, dual endothelin type A and AT_1_R blockade with sparsentan demonstrated additional antiproteinuric benefit compared with AT_1_R blockade alone. The exact mechanism of action linking *APOL1* high-risk genotypes to FSGS is the subject of intense research, and proposed pathways include alterations in pore-forming activity in the cell membrane, autophagy, and inflammation.^39^ Future research might elucidate how dual inhibition of angiotensin II type 1 and endothelin type A receptors interferes with these processes. Regardless of the mechanism, our findings indicate that sparsentan is likely effective in lowering proteinuria and consequently may be nephroprotective in patients with *APOL1* high-risk genotypes.

Based on a recent report, patients with one *APOL1* high-risk allele might be at a higher risk of developing FSGS and subsequent progression of CKD.^40^ In the subgroup of patients with a single *APOL1* high-risk allele, sparsentan had a similar effect as seen in the other genetic subgroups, with a trend toward a more pronounced antiproteinuric effect as compared with irbesartan.

Notably, across genetic subgroups, sparsentan not only led to greater proteinuria reduction but also to numerically higher rates of CR, a strong predictor of favorable long-term outcomes in FSGS.^20,41,42^ This finding further supports the nephroprotective potential of sparsentan in these forms of FSGS.

With respect to preservation of kidney function, there was a trend, albeit inconsistent, toward numerically less absolute decline in eGFR with sparsentan versus irbesartan in patients with gFSGS. While the absolute change in eGFR observed in patients with podocyte gene variants was greater than that in the overall DUPLEX population, the magnitude of eGFR decline among those with *COL4A3–5* variants and *APOL1* high-risk genotypes was generally consistent with observations in the overall DUPLEX population.^17^ Furthermore, the high degree of variability in eGFR decline is consistent with both the overall study population^17^ and large-scale analyses by the Proteinuria and Other Biomarkers as Endpoints for Clinical Trials in Kidney Disease Initiative.^42,23^ This international collaborative effort demonstrated that eGFR might not be a feasible clinical trial endpoint in FSGS, as its high variability requires large sample sizes and long duration of follow-up to confirm efficacy^42,43^ in this rare patient population. Nonetheless, despite the low number of patients and, consequently, low number of events, analyses of the proportion of patients reaching the composite kidney endpoint also favored treatment with sparsentan versus irbesartan in all genetic subgroups, consistent with findings in the overall study population.^17^ This observation is not only suggestive of greater nephroprotection with sparsentan but also indicates that observed greater antiproteinuric effect of sparsentan is not at the expense of kidney function.

Our observations supporting the efficacy of sparsentan in patients with gFSGS from DUPLEX are consistent with recent findings from experimental models of this subcategory of FSGS. For example, in *Trpc6* transgenic mice that recapitulate FSGS associated with activating variants in the *TRPC6* gene, sparsentan ameliorated podocyte injury and development of glomerulosclerosis, decreased albuminuria, reversed kidney inflammation, and promoted tissue repair.^44^ Similarly, sparsentan attenuated proteinuria, stabilized kidney function, and reduced glomerulosclerosis and tubulointerstitial fibrosis in the *Col4a3* knockout model of Alport syndrome.^45^ Moreover, these results are in accordance with abundant experimental evidence supporting the benefits of dual inhibition of AT_1_R and endothelin type A receptors and consequently sparsentan on podocyte viability, structural integrity, and function.^14,15,46^

There are several limitations to this study. First, we acknowledge that the patients with gFSGS in DUPLEX comprise a heterogenous group due to a variety of genetic variants that could theoretically impact treatment response. As a consequence, we have refrained from providing data summarizing findings in the overall gFSGS group. In addition, further covariates were not added to the MMRM model due to limited number of observations and convergence concerns. As a result, the MMRM model was not fully adjusted for all potential covariates (*e.g*., baseline patient characteristics). Owing to the small sample size in each genetic subgroup, the result has limited analytic power, which constrains our ability to draw definitive conclusions and assess more specific subgroups (*e.g*., individual podocyte variants and abnormalities in different collagen *α*-chains). Finally, the pathogenicity of genetic variants was determined using standards set by PreventionGenetics according to the American College of Medical Genetics and Genomics guidelines.^21^ While these standards are rigorous and widely accepted, there remains a possibility of misclassifying variants, although expected to be a low percentage. Despite these limitations, the study is notable for being the first to specifically assess the efficacy of sparsentan in patients with different forms of gFSGS, and, to our knowledge, the first long-term interventional trial in patients with variants in podocyte-specific genes, which are historically thought to be unresponsive to conventional therapies including nephroprotective therapies and immunosuppressants.

In summary, consistent with findings from the full DUPLEX trial^17^ cohort, we observed rapid and sustained proteinuria reductions with sparsentan compared with maximum dose irbesartan over approximately 2 years in all subtypes of gFSGS, including those due to monogenic variants in genes coding for podocyte proteins, variants in *COL4A3–5,* and *APOL1* high-risk genotypes. These findings suggest that the effect of sparsentan on proteinuria reduction and potential nephroprotection is preserved even in patients with genetic forms of FSGS, who are often resistant to other therapeutic interventions.

## Supplementary Material

## Data Availability

Original data generated for the study will be made available on reasonable request to the corresponding author. Data Type: Clinical Trial Data. Reason for Restricted Access: Travere is committed to data transparency and sharing data collected in completed and published Phase 3 clinical trials, observational trials, and post-marketing studies to further research while ensuring that patient privacy is protected. Pertinent individual patient-level data that underlie the results reported in a manuscript may be made available after deidentification. Relevant information may include redacted study protocol and redacted clinical study report. Requests for clinical trial data, including language stating its intended use, should be directed to datarequest@travere.com. If approved, the requested information will be provided to the requestor after signing a data access agreement. Requests can be made following completion of the study and full publication of the study data in a peer reviewed journal for up to 36 months following its publication. Travere reserves the right to decline or recommend modifications to a request if it does not comply with the data sharing policy or if it is determined that the request is made by a biased source.

## Declarative Statements

This study includes clinical experimentation and received Institutional Review Board or Ethics Committee approval. All patients provided written informed consent. This study includes clinical experimentation and complies with the Declaration of Helsinki.

## Supplemental Material

Supplemental Table 1. Nephrotic syndrome/FSGS gene panel (*N*=73).

Supplemental Table 2. *COL4A3–5* variants.

Supplemental Table 3. Geometric least squares mean percent change from baseline in UPCR at each study visit by genetic subgroup (95% CI).

Supplemental Table 4. General and baseline characteristics of *APOL1* heterozygous group.

Supplemental Figure 1. Effect of sparsentan compared with irbesartan on UPCR in patients with *NPHS2* variants.
