# Supplementary material for: Antiproteinuric Effect of Sparsentan in Patients with Genetic-Associated FSGS Enrolled in the DUPLEX Trial
Source: Clin J Am Soc Nephrol. 2025 Dec 23;21(4):605–14. doi: 10.2215/CJN.0000000948 (PMC13065193; doi:10.2215/CJN.0000000948)
Supplement: Supplementary file 1 [file cjasn-21-605-s001.pdf]

ASN American Society of Nephrology JASN Journal of the American Society of Nephrology KIDNEY360 Accessing Our World From Every Angle CJASN Clinical Journal of the American Society of Nephrology

ASN Journal Disclosure Form

As per ASN journal policy, I have disclosed any financial relationships or commitments I have held in the past 36 months as included below. I have listed my Current Employer below to indicate there is a relationship requiring disclosure. If no relationship exists, my Current Employer is not listed.

P. Bedard reports the following:
Consultancy: Travere Therapeutics, Inc.

I understand that the information above will be published within the journal article, if accepted, and that failure to comply and/or to accurately and completely report the potential financial conflicts of interest could lead to the following: 1) Prior to publication, article rejection, or 2) Post-publication, sanctions ranging from, but not limited to, issuing a correction, reporting the inaccurate information to the authors' institution, banning authors from submitting work to ASN journals for varying lengths of time, and/or retraction of the published work.

Name: Patricia W. Bedard
Manuscript ID: CJASN-2025-001069R1
Manuscript Title: ANTIPROTEINURIC EFFECT OF SPARSENTAN IN PATIENTS WITH GENETIC-ASSOCIATED FOCAL SEGMENTAL GLOMERULOSCLEROSIS ENROLLED IN THE DUPLEX TRIAL.
Date of Completion: October 17, 2025
Disclosure Updated Date: October 17, 2025

ASN American Society of Nephrology JASN Journal of the American Society of Nephrology KIDNEY360 Accessing Our World From Every Angle CJASN Clinical Journal of the American Society of Nephrology

ASN Journal Disclosure Form

As per ASN journal policy, I have disclosed any financial relationships or commitments I have held in the past 36 months as included below. I have listed my Current Employer below to indicate there is a relationship requiring disclosure. If no relationship exists, my Current Employer is not listed.

W. Gong reports the following:
Employer: Travere Therapeutics; and Ownership Interest: Travere Therapeutics.

I understand that the information above will be published within the journal article, if accepted, and that failure to comply and/or to accurately and completely report the potential financial conflicts of interest could lead to the following: 1) Prior to publication, article rejection, or 2) Post-publication, sanctions ranging from, but not limited to, issuing a correction, reporting the inaccurate information to the authors' institution, banning authors from submitting work to ASN journals for varying lengths of time, and/or retraction of the published work.

Name: Wu Gong
Manuscript ID: CJASN-2025-001069R1
Manuscript Title: Antiproteinuric effect of Sparsentan in patients with genetic-associated focal segmental glomerulosclerosis enrolled in the duplex trial
Date of Completion: October 21, 2025
Disclosure Updated Date: June 4, 2025

ASN American Society of Nephrology JASN Journal of the American Society of Nephrology KIDNEY360 Accessing Our World From Every Angle CJASN Clinical Journal of the American Society of Nephrology

ASN Journal Disclosure Form

As per ASN journal policy, I have disclosed any financial relationships or commitments I have held in the past 36 months as included below. I have listed my Current Employer below to indicate there is a relationship requiring disclosure. If no relationship exists, my Current Employer is not listed.

A. Gruber reports the following:
Employer: PreventionGenetics, part of Exact Sciences; and Ownership Interest: Exact Sciences.

I understand that the information above will be published within the journal article, if accepted, and that failure to comply and/or to accurately and completely report the potential financial conflicts of interest could lead to the following: 1) Prior to publication, article rejection, or 2) Post-publication, sanctions ranging from, but not limited to, issuing a correction, reporting the inaccurate information to the authors' institution, banning authors from submitting work to ASN journals for varying lengths of time, and/or retraction of the published work.

Name: Angela J Gruber
Manuscript ID: CJASN-2025-001069R1
Manuscript Title: Antiproteinuric effect of Sparsentan in patients with genetic-associated focal segmental glomerulosclerosis enrolled in the duplex trial
Date of Completion: October 21, 2025
Disclosure Updated Date: October 21, 2025

ASN
American Society of Nephrology
JASN
Journal of the American Society of Nephrology
KIDNEY360
Accessing Our World From Every Angle
CJASN
Clinical Journal of the American Society of Nephrology

ASN Journal Disclosure Form

As per ASN journal policy, I have disclosed any financial relationships or commitments I have held in the past 36 months as included below. I have listed my Current Employer below to indicate there is a relationship requiring disclosure. If no relationship exists, my Current Employer is not listed.

J. Inrig reports the following:
Employer: Travere Therapeutics; Ownership Interest: Travere Therapeutics; and Advisory or Leadership Role: Travere Therapeutics.

I understand that the information above will be published within the journal article, if accepted, and that failure to comply and/or to accurately and completely report the potential financial conflicts of interest could lead to the following: 1) Prior to publication, article rejection, or 2) Post-publication, sanctions ranging from, but not limited to, issuing a correction, reporting the inaccurate information to the authors' institution, banning authors from submitting work to ASN journals for varying lengths of time, and/or retraction of the published work.

Name: Jula K. Inrig
Manuscript ID: CJASN-2025-001069R1
Manuscript Title: Antiproteinuric effect of Sparsentan in patients with genetic-associated focal segmental glomerulosclerosis enrolled in the duplex trial
Date of Completion: November 3, 2025
Disclosure Updated Date: November 3, 2025

ASN American Society of Nephrology
JASN Journal of the American Society of Nephrology
KIDNEY360 Accessing Our World From Every Angle
CJASN Clinical Journal of the American Society of Nephrology

ASN Journal Disclosure Form

As per ASN journal policy, I have disclosed any financial relationships or commitments I have held in the past 36 months as included below. I have listed my Current Employer below to indicate there is a relationship requiring disclosure. If no relationship exists, my Current Employer is not listed.

R. Komers reports the following:
Employer: Travere Therapeutics; San Diego, CA ; 3611 Valley Centre Drive, Suite 300; San Diego, CA 92130;; Ownership Interest: Travere Therapeutics; and Patents or Royalties: Travere Therapeutics.

I understand that the information above will be published within the journal article, if accepted, and that failure to comply and/or to accurately and completely report the potential financial conflicts of interest could lead to the following: 1) Prior to publication, article rejection, or 2) Post-publication, sanctions ranging from, but not limited to, issuing a correction, reporting the inaccurate information to the authors' institution, banning authors from submitting work to ASN journals for varying lengths of time, and/or retraction of the published work.

Name: Radko Komers
Manuscript ID: CJASN-2025-001069R1
Manuscript Title: Antiproteinuric effect of Sparsentan in patients with genetic-associated focal segmental glomerulosclerosis enrolled in the Duplex trial
Date of Completion: October 21, 2025
Disclosure Updated Date: October 21, 2025

ASN American Society of Nephrology · JASN Journal of the American Society of Nephrology · KIDNEY360 Accessing Our World From Every Angle · CJASN Clinical Journal of the American Society of Nephrology

ASN Journal Disclosure Form

As per ASN journal policy, I have disclosed any financial relationships or commitments I have held in the past 36 months as included below. I have listed my Current Employer below to indicate there is a relationship requiring disclosure. If no relationship exists, my Current Employer is not listed.

J. Lai Yee reports the following:
Employer: University of Michigan

I understand that the information above will be published within the journal article, if accepted, and that failure to comply and/or to accurately and completely report the potential financial conflicts of interest could lead to the following: 1) Prior to publication, article rejection, or 2) Post-publication, sanctions ranging from, but not limited to, issuing a correction, reporting the inaccurate information to the authors' institution, banning authors from submitting work to ASN journals for varying lengths of time, and/or retraction of the published work.

Name: Jennifer Lai Yee
Manuscript ID: CJASN-2025-001069R1
Manuscript Title: Antiproteinuric effect of Sparsentan in patients with genetic-associated focal segmental glomerulosclerosis enrolled in the duplex trial
Date of Completion: October 21, 2025
Disclosure Updated Date: October 21, 2025

ASN
American Society of Nephrology
JASN
Journal of the American Society of Nephrology
KIDNEY360
Accessing Our World From Every Angle
CJASN
Clinical Journal of the American Society of Nephrology

ASN Journal Disclosure Form

As per ASN journal policy, I have disclosed any financial relationships or commitments I have held in the past 36 months as included below. I have listed my Current Employer below to indicate there is a relationship requiring disclosure. If no relationship exists, my Current Employer is not listed.

M. Rheault reports the following:
Employer: University of Minnesota; Consultancy: Otsuka, Calliditas, ELOXX, Vifor; Ownership Interest: Protolabs, Microsoft; Research Funding: Chinook/Novartis, Travere, River 3 Renal, Aurinia, Dimerix; Patents or Royalties: Wolters Kluwer-Royalties for Textbook of Glomerulonephritis as editor; and Advisory or Leadership Role: Alport Syndrome Foundation Medical Advisory Board; NephJC (501c3) Board of Directors; all unpaid.

I understand that the information above will be published within the journal article, if accepted, and that failure to comply and/or to accurately and completely report the potential financial conflicts of interest could lead to the following: 1) Prior to publication, article rejection, or 2) Post-publication, sanctions ranging from, but not limited to, issuing a correction, reporting the inaccurate information to the authors' institution, banning authors from submitting work to ASN journals for varying lengths of time, and/or retraction of the published work.

Name: Michelle N. Rheault
Manuscript ID: CJASN-2025-001069R1
Manuscript Title: Antiproteinuric effect of Sparsentan in patients with genetic-associated focal segmental glomerulosclerosis enrolled in the duplex trial
Date of Completion: November 3, 2025
Disclosure Updated Date: November 3, 2025

ASN Journal Disclosure Form

As per ASN journal policy, I have disclosed any financial relationships or commitments I have held in the past 36 months as included below. I have listed my Current Employer below to indicate there is a relationship requiring disclosure. If no relationship exists, my Current Employer is not listed.

H. Trachtman reports the following:
Employer: University of Michigan Adjunct Professor of Pediatrics; RenalStrategies LLC; Consultancy: Otsuka (DSMB Chair), Bristol Meyers Squibb (inactive), Chemocentryx (DMSB) (inactive), Goldfinch Bio (inactive), Travere Therapeutics, Natera (RenaSight) (inactive), Angion (inactive), Akebia, Walden, Aclipse, Akebia, Alentis, Apellis, Hi-Bio (Biogen), Boehringer-Ingelheim, PhaseV, Maze Therapeutics, Novartis, Alexion/Astra Zeneca, Eloxx Pharmaceuticals, Dimerix, Vera, OneFourBio, NephCure Kidney International, ProKidney (inactive) Kaneka (inactive), Astellas (inactive), Angion (inactive),; Ownership Interest: Aclipse; PhaseV; Honoraria: Attendance at glomerular disease panels organized by Reata and Astellas, Advisory Board for Otsuka, Travere Therapeutics, Boehringer-Ingelheim, Maze Therapeutics, Vera; and Advisory or Leadership Role: DSMB RIVUR Trial (completed) ; DSMB bumetanide trial (completed); Chair, DSMB, Otsuka trials; DSMB, ANCA vasculitis, Chemocentryx (completed); Steering Committee, Abatacept Trial, BMS (completed); DUPRO Steering Committee, Travere (completed); Steering Committee, Goldfinch Bio (inactive); ProKidney, Scientific Advisory Board (inactive); MEDCAC; KHI Board of Directors; Editorial Board, Pediatric Nephrology, Kidney360, and Glomerular Diseases; Editor-in-chief, Expert Opinion on Emerging Drugs.

I understand that the information above will be published within the journal article, if accepted, and that failure to comply and/or to accurately and completely report the potential financial conflicts of interest could lead to the following: 1) Prior to publication, article rejection, or 2) Post-publication, sanctions ranging from, but not limited to, issuing a correction, reporting the inaccurate information to the authors' institution, banning authors from submitting work to ASN journals for varying lengths of time, and/or retraction of the published work.

Name: Howard Trachtman
Manuscript ID: CJASN-2025-001069R1
Manuscript Title: Antiproteinuric effect of Sparsentan in patients with genetic-associated focal segmental glomerulosclerosis enrolled in the duplex trial,
Date of Completion: November 3, 2025
Disclosure Updated Date: October 23, 2025
