# Supplementary material for: Antiproteinuric Effect of Sparsentan in Patients with Genetic-Associated FSGS Enrolled in the DUPLEX Trial
Source: Clin J Am Soc Nephrol. 2025 Dec 23;21(4):605–14. doi: 10.2215/CJN.0000000948 (PMC13065193; doi:10.2215/CJN.0000000948)
Supplement: Supplementary file 2 [file cjasn-21-605-s002.pdf]

SUPPLEMENT TO:

ANTIPROTEINURIC EFFECT OF SPARSENTAN IN PATIENTS WITH GENETIC-ASSOCIATED FOCAL SEGMENTAL GLOMERULOSCLEROSIS ENROLLED IN THE DUPLEX TRIAL

Yee J, et al.

TABLE OF CONTENTS

Supplemental Table 1. Nephrotic syndrome/focal segmental glomerulosclerosis gene panel (N=73) 1

Supplemental Table 2. *COL4A3-5* variants 2

Supplemental Table 3. Geometric least squares mean percent change from baseline in UPCR at each study visit by genetic subgroup (95% CI) 3

Supplemental Table 4. General and baseline characteristics of *APOL1* heterozygous group.......................... 4

Supplemental Figure 1. Effect of sparsentan compared to irbesartan on UPCR in patients with *NPHS2* variants 5

Supplemental Table 1. Nephrotic syndrome/focal segmental glomerulosclerosis gene panel (N=73)*

ACTN4	*DLC1*	*MAGI2*	*SMARCAL1*
ANKFY1	*EMP2*	*MYH9*	*TBC1D8B*
ANLN	*FAT1*	*MYO1E*	*TNS2*
APOL1	*GAPVD1*	*NEU1*	*TP53RK*
ARHGAP24	*GON7*	*NFKB2*	*TPRKB*
ARHGDIA	*INF2*	*NPHS1*	*TRIM8*
AVIL	*ITGA3*	*NPHS2*	*TRPC6*
CD2AP	*ITGB4*	*NUP107*	*TTC21B*
CDK20	*ITSN1*	*NUP133*	*WDR4*
COL4A3	*ITSN2*	*NUP160*	*WDR73*
COL4A4	*KANK1*	*NUP205*	*WT1*
COL4A5	*KANK2*	*NUP93*	*XPO5*
COL4A6	*KANK4*	*OSGEP*	*YRDC*
COQ2	*KAT2B*	*PAX2*	
COQ6	*KIRREL1*	*PDSS2*	
COQ8B	*LAGE3*	*PLCE1*	
CRB2	*LAMA5*	*PODXL*	
CUBN	*LAMB2*	*PTPRO*	
DAAM2	*LMX1B*	*SCARB2*	
DGKE	*MAFB*	*SGPL1*	

ACTN4, actinin α4; *ANKFY1*, ankyrin repeats- and FYVE domain-containing protein 1; *ANLN*, actin-binding protein anillin; *APOL1*, apolipoprotein L1; *ARHGAP24*, rho GTPase-activating protein 24; *ARHGDIA*, rho GDP-dissociation inhibitor α; *AVIL*, advillin; *CD2AP*, CD2 associated protein; *CDK20,* cyclin-dependent kinase 20; *COL4A3*, collagen type IV (COL4) α3; *COL4A4*, collagen type IV (COL4) α4; *COL4A5*, collagen type IV (COL4) α5; *COL4A6*, collagen type IV (COL4) α6; *COQ2,* coenzyme Q2; *COQ6,* coenzyme Q6; *COQ8B,* coenzyme Q8β; *CRB2,* crumbs cell polarity complex component 2; *CUBN,* cubilin; *DAAM2*, disheveled-associated activator of morphogenesis 2; *DGKE,* diacylglycerol kinase ε; *DLC1*, rho GTPase-activating protein; *EMP2,* epithelial membrane protein 2; *FAT1,* fat atypical cadherin 1; FYVE, Fab1, YOTB/ZK632.12, Vac1, early endosome antigen 1). *GAPVD1*, GTPase-activating protein and VPS9 domains 1; GDP, guanosine diphosphate; *GON7*, GON7 subunit of KEOPS complex; GTP, guanosine triphosphate; *INF2*, inverted formin 2; *ITGA3*, integrin α3; *ITGB4*, integrin β4; *ITSN1,* intersectin 1; *ITSN2,* intersectin 2; *KANK1,* KN motif- and ankyrin repeat domain-containing protein 1; *KANK2,* KN motif- and ankyrin repeat domain-containing protein 2; *KANK4,* KN motif- and ankyrin repeat domain-containing protein 4; *KAT2B*, lysine acetyltransferase 2B; KEOPS, kinase, putative endopeptidase, and other proteins of small size; *KIRREL1*, kirre-like nephrin family adhesion molecule 1; KN, KANK N-terminal; *LAGE3,* L antigen family member 3; *LAMA5*, laminin α5; *LAMB2,* laminin β2; *LMX1B*, LIM homeobox transcription factor 1β; MAF, musculoaponeurotic fibrosarcoma; *MAFB,* MAF bZIP transcription factor B; *MAGI2,* membrane-associated guanylate kinase WW and PDZ domains-containing protein 2; *MYH9,* myosin heavy chain 9; *MYO1E,* myosin IE; *NEU1,* neuraminidase 1; *NFKB2,* nuclear factor κB subunit 2; *NPHS1*, nephrin; *NPHS2*, podocin; *NUP107,* nucleoporin 107-kD; *NUP133,* nucleoporin 133-kD; *NUP160,* nucleoporin 160-kD; *NUP205,* nucleoporin 205-kD; *NUP93,* nucleoporin 93-kD; *OSGEP,* O-sialoglycoprotein endopeptidase; *PAX2,* paired box gene 2; *PDSS2,* prenyl diphosphate synthase subunit 2; PDZ, postsynaptic density-95/discs large/zona occludens-1; *PLCE1,* phospholipase C ε1; *PODXL,* podocalyxin-like protein; *PTPRO,* protein-tyrosine phosphatase receptor-type O; *SCARB2*, scavenger receptor class B member 2; *SGPL1,* sphingosine-1-phosphate lyase 1; *SMARCAL1,* SWI/SNF-related matrix-associated actin-dependent regulator of chromatin subfamily A-like protein 1; SWI/SNF, switch/sucrose non-fermentable; TBC, Tre-2/Bub2/Cdc16; *TBC1D8B,* TBC1 domain family member 8B; *TNS2,* tensin 2; *TP53RK,* TP53-regulating kinase; *TPRKB*, TP53RK-binding protein; *TRIM8,* tripartite motif-containing protein 8; *TRPC6*, transient receptor potential cation channel subfamily C member 6; *TTC21B,* tetratricopeptide repeat domain-containing protein 21B; WD, W-dipeptide; *WDR4,* WD repeat-containing protein 4; *WDR73,* WD repeat-containing protein 73; *WT1*, WT1 transcription factor; *XPO5,* exportin 5; *YRDC*, YRDC domain-containing protein.

*Podocalyxin (PODXL) was added to the nephrotic syndrome/focal segmental glomerulosclerosis gene panel by PreventionGenetics.

Supplemental Table 2. *COL4A3-5* variants

	COL4A3	***COL4A4***	***COL4A5***
**n**	12	9	4
**Sex ratio**	2 male:10 female	2 male:7 female	2 male:2 female
**Genotype and treatment**			
Heterozygous	12 (sparsentan, n=6; irbesartan n=6)	8 (sparsentan, n=3; irbesartan, n=5)	2 (sparsentan, n=0; irbesartan, n=2)
Double heterozygous in cis	-	1* (sparsentan, n=1)	-
Hemizygous	-	-	2 (sparsentan, n=1; irbesartan, n=1)

COL4A3, collagen type IV (COL4) α3; *COL4A4*, collagen type IV (COL4) α4; *COL4A5*, collagen type IV (COL4) α5; *COL4A3-5*, collagen type IV (COL4) α3, α4, and α5.
*Patient has *COL4A4* c.2320G>C (p.Gly774Arg) and c.4394G>A (p.Gly1465Asp), which are typically reported to be in cis.

Supplemental Table 3. Geometric least squares mean percent change from baseline in UPCR at each study visit by genetic subgroup (95% CI)

**Podocyte gene variants**										
**Week**	6	12	24	36	48	60	72	84	96	108
**n**										
Sparsentan	13	13	12	12	11	11	10	10	9	9
Irbesartan	17	15	14	15	14	10	12	12	13	11
**Geometric LS mean change from baseline in UPCR, % (95% CI)**										
Sparsentan	–52.5 (–66.3 to –33.0)	–52.8 (–66.5 to –33.5)	–55.2 (–68.5 to –36.4)	–63.3 (–74.3 to –47.7)	–47.6 (–63.7 to –24.5)	–46.0 (–62.8 to –21.6)	–52.5 (–67.7 to –30.1)	–46.8 (–64.1 to –21.1)	–40.7 (–60.4 to –11.1)	–49.1 (–66.3 to –23.1)
Irbesartan	–20.1 (–40.6 to 7.6)	–24.8 (–44.5 to 1.9)	–28.9 (–47.8 to –3.1)	–35.4 (–52.5 to –12.1)	–23.2 (–44.0 to 5.5)	–25.8 (–47.3 to 4.5)	–20.6 (–43.3 to 11.2)	–32.1 (–51.6 to –4.6)	–29.7 (–49.8 to –1.5)	–27.5 (–49.0 to 3.0)
***NPHS2* variants**										
**Week**	6	12	24	36	48	60	72	84	96	108
**n**										
Sparsentan	9	9	9	9	9	9	8	8	7	7
Irbesartan	5	4	4	6	5	2	4	3	4	3
**Geometric LS mean change from baseline in UPCR, % (95% CI)**										
Sparsentan	–54.7 (–70.0 to –31.7)	–53.8 (–69.4 to –30.3)	–55.3 (–70.4 to –32.6)	–66.3 (–77.6 to –49.1)	–48.2 (–65.6 to –21.8)	–48.9 (–66.1 to –22.9)	–53.1 (–69.3 to –28.5)	–51.2 (–68.3 to –24.9)	–40.8 (–62.0 to –7.7)	–44.9 (–65.0 to –13.3)
Irbesartan	–26.9 (–57.3 to 25.0)	–32.9 (–61.7 to 17.6)	–42.5 (–67.1 to 0.6)	–47.5 (–68.8 to –11.4)	–24.9 (–56.0 to 28.2)	–35.9 (–66.1 to 21.2)	–24.2 (–57.7 to 35.8)	–52.6 (–74.4 to –11.9)	–51.3 (–73.3 to –11.0)	–50.0 (–73.6 to –5.1)
***COL4A3-5* variants**										
**Week**	6	12	24	36	48	60	72	84	96	108
**n**										
Sparsentan	11	10	11	11	10	10	10	10	10	9
Irbesartan	13	14	13	12	11	10	10	11	10	10
**Geometric LS mean change from baseline in UPCR, % (95% CI)**										
Sparsentan	–58.8 (–72.2 to –39.0)	–56.7 (–70.9 to –35.5)	–59.2 (–72.5 to –39.6)	–59.3 (–72.5 to –39.7)	–59.7 (–73.0 to –39.9)	–65.8 (–77.2 to –48.8)	–60.7 (–73.9 to –41.0)	–59.4 (–73.1 to –38.9)	–55.3 (–70.4 to –32.6)	–48.0 (–65.8 to –20.8)
Irbesartan	–19.6 (–43.7 to 14.6)	–32.3 (–52.4 to –3.9)	–19.0 (–43.2 to 15.7)	–25.1 (–47.9 to 7.7)	–35.1 (–55.2 to –5.9)	–25.1 (–48.8 to 9.7)	–30.8 (–53.0 to 1.9)	–29.2 (–51.9 to 4.2)	–6.3 (–36.9 to 39.1)	–34.1 (–55.9 to –1.6)
***APOL1* high-risk genotypes**										
**Week**	6	12	24	36	48	60	72	84	96	108
**n**										
Sparsentan	7	8	8	7	7	6	6	6	5	6
Irbesartan	5	5	5	5	4	4	3	3	3	3
**Geometric LS mean change from baseline in UPCR, % (95% CI)**										
Sparsentan	–50.7 (–76.7 to 4.4)	–57.1 (–79.5 to -9.9)	–50.7 (–76.5 to 3.5)	–51.6 (–77.0 to 1.9)	–53.7 (–78.1 to –2.0)	–60.4 (–81.5 to –15.2)	–48.2 (–76.1 to 12.3)	–54.5 (–79.2 to –0.3)	–51.1 (–78.0 to 8.8)	–55.0 (–85.2 to 36.7)
Irbesartan	–26.9 (–72.3 to 93.4)	–23.3 (–71.0 to 102.8)	–38.5 (–76.8 to 62.5)	–37.7 (–76.5 to 64.7)	–37.8 (–76.8 to 66.7)	–10.7 (–67.1 to 142.3)	–38.4 (–78.1 to 72.9)	–30.6 (–76.0 to 100.9)	–47.7 (–82.4 to 55.3)	–46.2 (–75.8 to 19.9)

APOL1, apolipoprotein L1; CI, confidence interval; *COL4A3-5*, collagen type IV (COL4) α3, α4, and α5; LS, least squares; *NPHS2*, podocin; UPCR, urine protein-to-creatinine ratio.

Supplemental Table 4. General and baseline characteristics of *APOL1* heterozygous group

	APOL1 heterozygous group		
	**Sparsentan (n=5)**	**Irbesartan (n=10)**	**Total (n=15)**
**Age at informed consent, mean (SD), years**	37 (21)	40 (20)	39 (20)
**Age group, n (%)**			
<18 years	0 (0)	2 (20)	2 (13)
≥18 years	5 (100)	8 (80)	13 (87)
**Sex, n (%)**			
Male	1 (20)	4 (40)	5 (33)
Female	4 (80)	6 (60)	10 (67)
**Race, n (%)**			
Asian	0 (0)	0 (0)	0 (0)
Black or African American	3 (60)	7 (70)	10 (67)
White	1 (20)	2 (20)	3 (20)
Other	1 (20)	2 (20)	3 (20)
**BMI group, n (%)**			
<27 kg/m^2	3 (60)	3 (30)	6 (40)
≥27 kg/m^2	2 (40)	7 (70)	9 (60)
**eGFR, mL/min/1.73 m^2**			
Mean (SD)*	63 (23)	67 (29)	65 (27)
Median (IQR)	65 (42, 83)	72 (42, 88)	71 (42, 88)
**UPCR, g/g**			
Mean (SD)	2.5 (1.1)	3.9 (2.3)	3.5 (2.0)
Median (IQR)	2.4 (2.0, 2.7)	3.5 (2.6, 4.0)	2.8 (2.4, 4.0)
**Geometric LS mean percent change from baseline in UPCR, %**			
36 weeks	−67.7	−36.4	
72 weeks	−58.6	−49.9	
108 weeks	−65.3	−44.9	
**Complete remission of proteinuria, n (%)**	1 (20)	0 (0)	1 (7)
**Composite kidney outcome (40% eGFR reduction, ESKD, death), n (%)**	1 (20)	2 (20)	3 (20)

APOL1, apolipoprotein L1; BMI, body mass index; eGFR, estimated glomerular filtration rate; ESKD, end-stage kidney disease; IQR, interquartile range; LS, least squares; SD, standard deviation; UPCR, urine protein-to-creatinine ratio.

Supplemental Figure 1. Effect of sparsentan compared to irbesartan on UPCR in patients with *NPHS2* variants.

Percent change from baseline in UPCR at each study visit in patients with *NPHS2* variants.

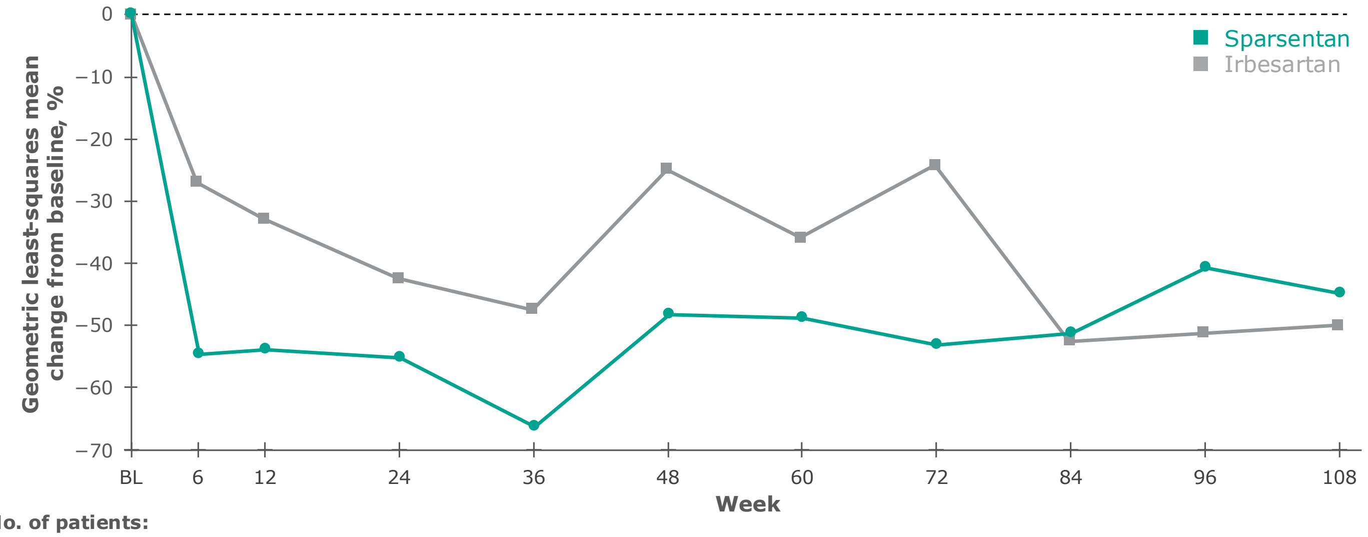

No. of patients:											
Irbesartan	6	5	4	4	6	5	2	4	3	4	3
Sparsentan	9	9	9	9	9	9	9	8	8	7	7

BL, baseline; UPCR, urine protein-to-creatinine ratio; *NPHS2*, podocin.
